# Creating a social virtual reality application for psychological research: A tutorial

**DOI:** 10.3758/s13428-025-02693-4

**Published:** 2025-06-04

**Authors:** Marius Rubo

**Affiliations:** Cognitive Psychology, Perception and Research Methods, Institute of Psychology, University of Bern, Bern, Switzerland

**Keywords:** Social virtual reality, Networked simulations, Social interactions, Experimental software

## Abstract

Social virtual reality (VR) setups allow two or more individuals to interact in a shared virtual environment while embodying computerized avatars. Such setups allow for detailed investigations into social-cognitive processes, can extend the functionality of existing single-user VR applications and can be used to design novel educational settings and psychotherapeutic treatments. While researchers may use commercially available social VR applications in addressing a range of research questions, an in-house software solution is typically developed when a research project requires more flexible experimental control or should conform to the highest data security standards. This tutorial demonstrates the construction of a social VR application based on an example software that mimics a VR setup but can be run and explored on an individual computer. Information flow emphasizes transparency to allow researchers to flexibly adapt the software to their own environment and research demands. The software is designed for use in controlled laboratory environments but can be extended for use in field research.

## Introduction

**Fig. 1:**
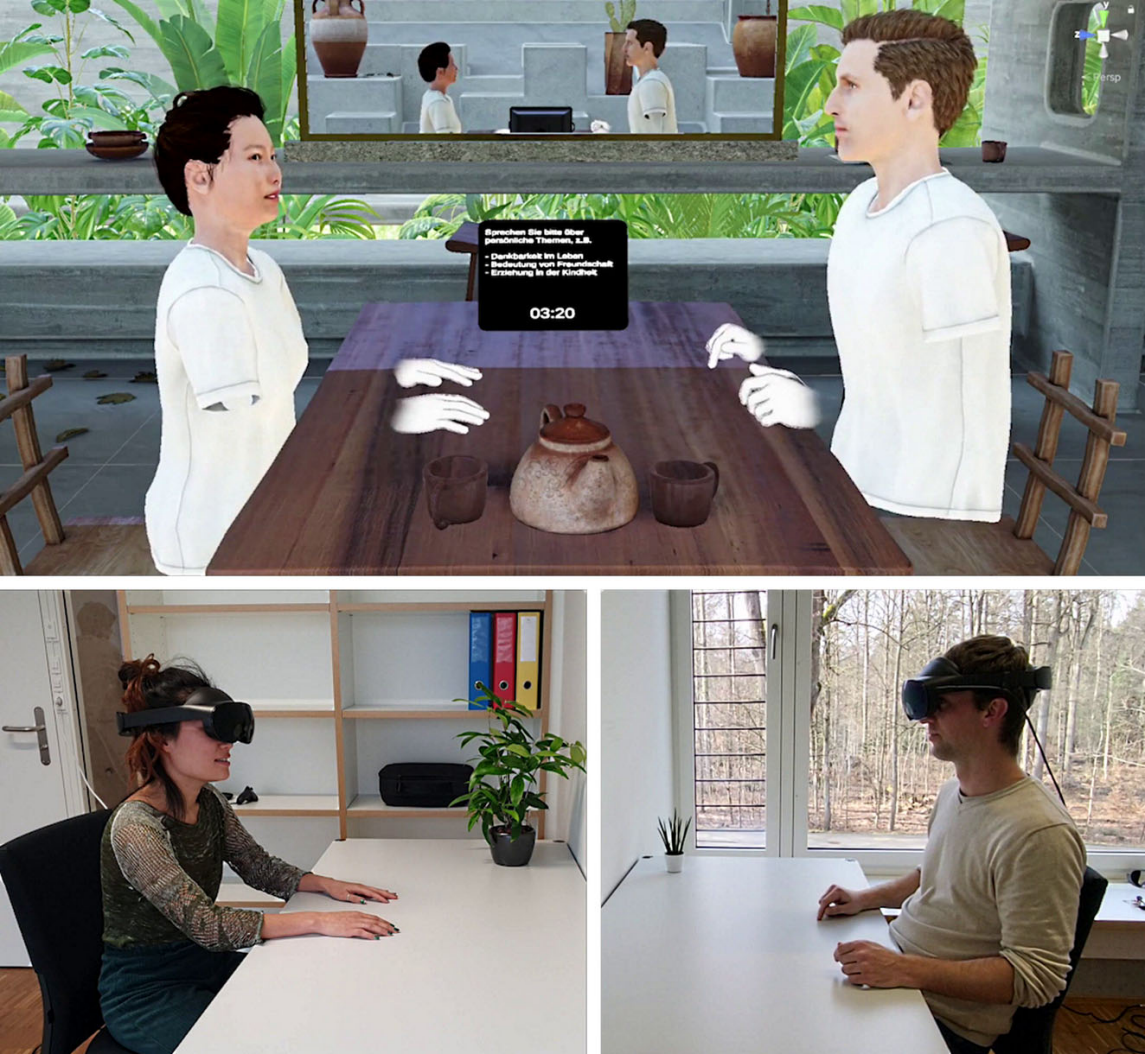
Social VR setup where two individuals embody avatars in a shared scenario (*top image*) while physically located in separate rooms (*bottom images*)

Virtual reality (VR) has been used in a range of basic and applied research fields such as clinical psychology and psychotherapy (Carl et al., 2019; Emmelkamp & Meyerbröker, 2021; Morina et al., 2021; Riva et al., 2019), education (Barteit et al., 2021; Radianti et al., 2020), language sciences (Peeters, 2019), bodily self-consciousness (Blanke, 2012; Kilteni et al., 2012) or neuro-rehabilitation (Khan et al., 2021). By allowing to exert precise experimental control over realistically displayed 3D stimuli, the technology can enhance researchers’ capabilities in attaining both internal and external validity in the same study (Miller et al., 2019; Pan et al., 2018). Across fields, the most common mode of using VR was a single-user setup where *one* participant viewed a 3D simulation in solitude, with experimenters or spectators seeing a representation of the VR user’s view on a computer screen. Although behavior in interactions with computer-controlled characters showed resemblance with human–human interactions (Dechant et al., 2017; Freeman et al., 2014; Dechant et al., 2021; Neumann et al., 2023; Pan et al., 2012, 2018; Rubo & Munsch, 2024; Schönbrodt & Asendorpf, 2011; Tarr et al., 2018) – indicating an at least partial suitability of such setups in investigating social-cognitive processes – researchers in several fields also investigate interactions between two or more real participants as they occupy the same virtual environment. Such *Social* VR setups (also termed *multi-user VR*, or *Metaverse* applications when embedded in a larger digital infrastructure) were already advocated as communication tools decades ago (Benford et al., 1995; Blanchard et al., 1990) but only gained more traction in behavioral research relatively recently. Modern installments of the technique can allow two or more individuals to embody relatively detailed computerized avatars which display facial expressions and eye gaze along with body and hand movements in real-time through dedicated sensors (see Fig. 1). Despite its artificiality, such environments were observed to elicit natural behavioral patterns as known from face-to-face interactions (Abdullah et al., 2021; Son & Rubo, 2025). Social VR was therefore contrasted with other distance communication tools such as videoconferencing, where the natural integration of speech and gaze behavior is often impaired (Abdullah et al., 2021; Bailenson, 2021).

Social VR may thus represent a viable research environment in many research fields which are interested in human–human interactions. In comparison with behavioral research in face-to-face interactions, social VR additionally allows to (1) more easily streamline assessments of behavioral metrics as they are built-in to the system and represented in a shared spatial reference frame, (2) exert more precise experimental control over objects and events in the shared environment and (3) investigate embodied interactions even when participants are physically located at a distance. By integrating the naturalness of face-to-face interactions within controlled laboratory setups, social VR may furthermore help to address the challenge of enhancing ecological validity in basic social attention and cognition research as previously proposed (Ojeda et al., 2014; Schilbach et al., 2013; Stangl et al., 2023). A range of research applications for social VR was previously demonstrated or envisioned (Ford et al., 2023; Lee, 2023; Riva et al., 2024). For instance, the technique was used to investigate how dyads integrate pointing gestures, eye gaze and grasp actions in navigating a collaborative task (Caruana et al., 2021; Nalepka et al., 2019) but was also used as a remote educational setting (Alfaisal et al., 2022; Di Natale et al., 2024a, b) or in mitigating social isolation (Thielbar et al., 2020).

Current social VR studies often assess behavior in participants using commercially available software applications (Han et al., 2023; Hennig-Thurau et al., 2023; McVeigh-Schultz et al., 2019). When a research question requires enhanced experimental control or flexibility in data collection, or if it entails strict data privacy demands which cannot be met using commercial software where data are transmitted to third party servers (Hoofnagle et al., 2018; Lin & Latoschik, 2022), researchers may instead develop an in-house social VR solution (Abdullah et al., 2021; Moser et al., 2020; Pan et al., 2017; Roth et al., 2018; Smith & Neff, 2018; Son & Rubo, 2025; Steed et al., 2022; Triandafilou et al., 2018). Such controlled setups can also allow to hold average end-to-end latency relatively low, which was measured at 48ms in our own setup (Son & Rubo, 2025) but above 100ms in commercial platforms (Cheng et al., 2022). Here I describe such a networked simulation and how it can be implemented into researchers’ own environments for a range of research contexts.

The tool is in use in our own laboratory where we investigated behavioral interaction patterns and the influence of anonymization on communication in dyads (Son & Rubo, 2025). Participants wore Meta Quest Pro head-mounted displays (HMDs) (https://www.meta.com) with built-in eye trackers, face tracker as well as hand and finger trackers. HMDs were connected to laptops to allow for higher-quality graphical quality. Dyads conversed on a range of topics (e.g., small-talk, more personal talk) while sitting on the same table in a virtual environment where their eye gaze, facial expressions and body movements were visible to the interaction partner (see Fig. 1). Participants in one experimental condition embodied avatars which resembled their physical appearance, while participants in the other condition embodied generic avatars, which, as the situation incorporated no physical contact between participants, entailed an anonymization of the interaction. Similar to face-to-face interactions, participants spent more time gazing towards their partner’s eye region while listening compared to while speaking. Participants furthermore spent more time gazing towards their partners’ eye region when ending compared to when beginning a speaking turn, again in line with normal face-to-face behavior. Although anonymization was found to elicit social disinhibition in other communication forms, anonymized communication in social VR was not associated with related behavioral markers such as louder speaking, more interrupting or more gaze towards the partner’s eye region. A possible explanation for intact social regulation in anonymized interactions in social VR is the possibility to engage in direct eye contact, which may facilitate psychological closeness even when experienced only in a computer-mediated form.

This article describes an open-source example software which can be used to design experimental setups as the ones used in our own laboratory but also a range of other setups which implement networked communication between VR users and systematic logging of behavioral metrics. The example software is designed to run on a set of computers or even a single computer with no need for VR hardware. Such a mock setup allows to more easily trace the tool’s information flow and to more conveniently implement and debug novel features. Guidelines are given to move the tool from its use in a mock environment to researchers’ existing VR infrastructure. The tutorial assumes knowledge on the setup of single-user VR environments outlined elsewhere (Jones et al., 2022; Spanlang et al., 2014; Vasser & Aru, 2020) but requires no knowledge in network communication and no advanced software development knowledge. Specifically, researchers may find the tool directly applicable to their own development of a social VR infrastructure if they (1) have previously worked with the Unity game engine (https://unity.com) or a similar 3D development environment, (2) have knowledge of the structure of code written in the C# language (3) have a conceptual understanding of installing virtual body illusions in VR and (4) have previously worked on the processing of moment-to-moment data streams as in eye tracking. The tutorial is thus most directly tailored to researchers who have already conducted studies involving virtual body illusions with eye-tracking in VR and now plan to implement multi-user setups in their environment.

## Software

### Architecture

In social VR as well as in other networked simulations, the experience of viewing and acting in a *shared* environment may be seen as an illusion as technically, every user is confronted with a unique simulation created on their respective computer. Commonalities between simulations are actively created and maintained throughout the interaction using specialized software modules often referred to as network synchronization code or *netcode* for short. Shared environments are typically available to all computers prior to starting a networking connection as these are shipped with the software. By contrast, users’ behavior as well as other events are continuously transmitted between computers and each computer is responsible for updating their simulation accordingly.

**Fig. 2:**
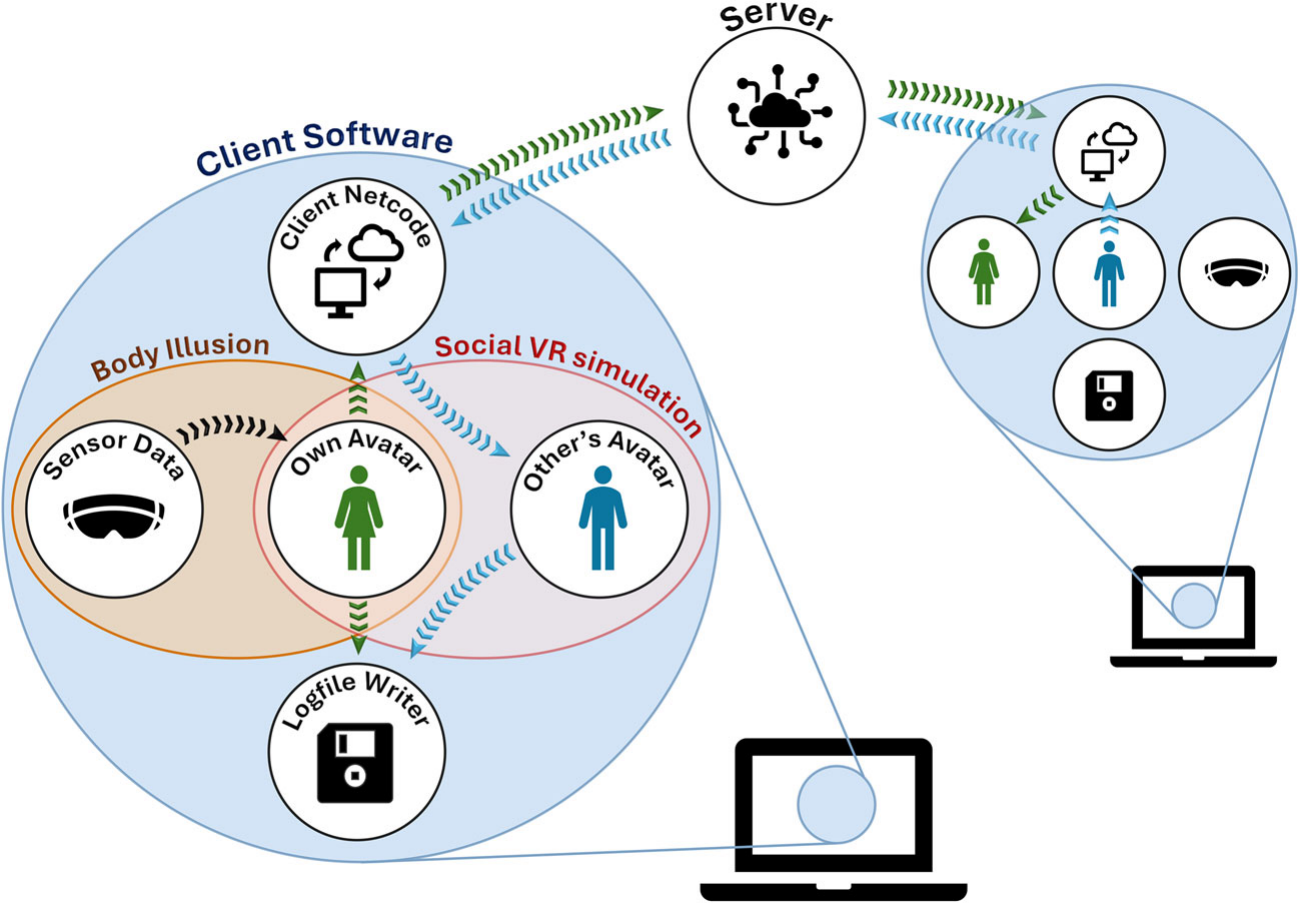
Architecture of the example software. Each client software maintains its own social VR simulation based on the user’s own sensor data as well other users’ behavioral data, which are relayed on the server

Figure 2 summarizes the architecture of the networked setup described here. The example software is available at https://github.com/mariusrubo/SocialVRExample. While each user’s *client software* may communicate directly with each other in some networked setups, a particularly robust configuration includes a server which organizes data transfer among clients and can authoritatively decide upon specific events. The server software does not need to run on a separate computer but can run on one of the client computers along with the client software, forming a host. Fundamentals of networked communication are covered in detail elsewhere (Steed & Oliveira, 2009) but are not required to follow or extend the provided example software. At the lowest level, communication between computers is handled by a the open-source netcode solution *Fish-Net* (https://github.com/FirstGearGames/FishNet/). Network communication using this tool requires no data transfer to third-party services, allowing for full control over the information flow.

At its core, the client software includes a body illusion setup where an avatar’s behavior is driven by real-time sensor data. This module corresponds to single-user body illusion setups used in research on bodily consciousness and other research fields. When the software is executed with only the body illusion module enabled, a user can already immerse in the virtual scene, look around and may see her own representation in a virtual mirror – but there is no-one else with her in the virtual environment. In the example software, avatars are positioned so that their eyes match the user’s eyes in order to allow for direct eye contact with oneself in a mirror as well as with other users.

Network functionality is implemented as an additional module: the client software’s netcode component initiates communication with the server and continuously sends behavioral data used in the body illusion. It furthermore instantiates avatars representing other users and continuously updates their states as it receives behavioral data from them via the server. In combination, the visualization of one’s own and other users’ avatars in a scene forms the social VR simulation depicted in the top panel of Fig. 1. Data from the simulation are stored to disk for later analyses using logfile writing modules. The server carries out comparatively fewer tasks in this setup, mostly relaying data between clients and organizing the assignment of seats on a table.

Note that in the setup described here, each client software autonomously controls movements of the participant’s own virtual avatar based on incoming data from the HMD’s sensors, even when it is connected to a server. In contrast to such a client-authoritative design, commercial networked software sometimes implements server-authoritative designs where input from a client is first processed by the server before being implemented in controlling character movements on the client. Such setups can better ensure simulation consistency in specific forms of interactions (e.g., collisions) and prevent irregular movements on the client side but add an additional delay between user input and simulation updates which may cause simulator sickness in VR (Dużmańska et al., 2018). The problem can be addressed by implementing parallel streams of data processing on the client and on the server which are reconciled when deviations occur (so-called client-side prediction with rollback and reconciliation). For a majority of applications within behavioral research, client-authoritative designs as used here may be an appropriate and light-weight software design which can be easily complemented with individual elements of server-authoritative events as needed. Participants in our own study (Son & Rubo, 2025) experienced moderate to high levels of presence and social presence as well as overall low levels of cybersickness similar to studies which involve no networked data processing. These findings are unsurprising since network delays never resulted in additional delays between participants’ own movements and image generation. Individual server-authoritative events such as updates in the display of instruction texts in the virtual environment did not affect the direct relationship between sensor input and image generation.

### Getting started

**Fig. 3:**
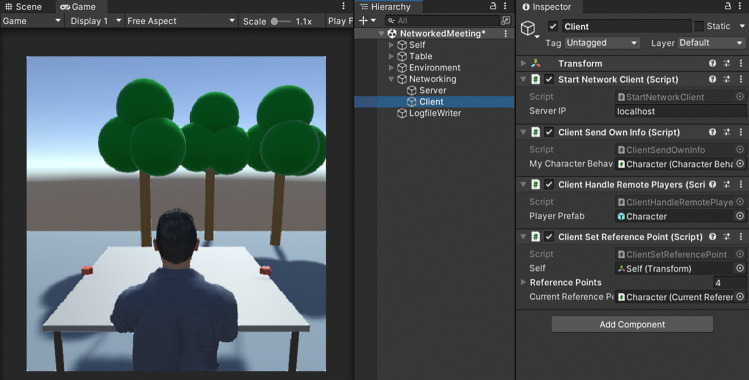
The example software’s scene. Character control, networking functionality and logfile writers are implemented as separate modules which are attached to individual objects. When all objects are activated, the program acts as a host (i.e., acting both as the server and one client). Standalone server, client or host programs are created by activating or deactivating respective objects in the scene before building the software

The example software is a self-contained Unity project (for Unity version 2021.3.2 or above) and requires no additional downloads and no VR hardware. It is designed to quickly enable individual researchers to set up a networked simulation using relatively simple and effective methods. Open the scene *NetworkedMeeting* to find the setup shown in Fig. 3. Control of the user’s own character is organized in the object *Self* which mimics the streaming of sensor data from VR hardware. Networking and logfile writing modules are attached to their own objects. When starting the scene, the character will sit on a table and gaze where the user points the mouse cursor. For the program to note down logfiles, create a folder ‘SocialVR’ with a subfolder ‘Data’ in your computer’s ‘Documents’ folder.

**Fig. 4:**
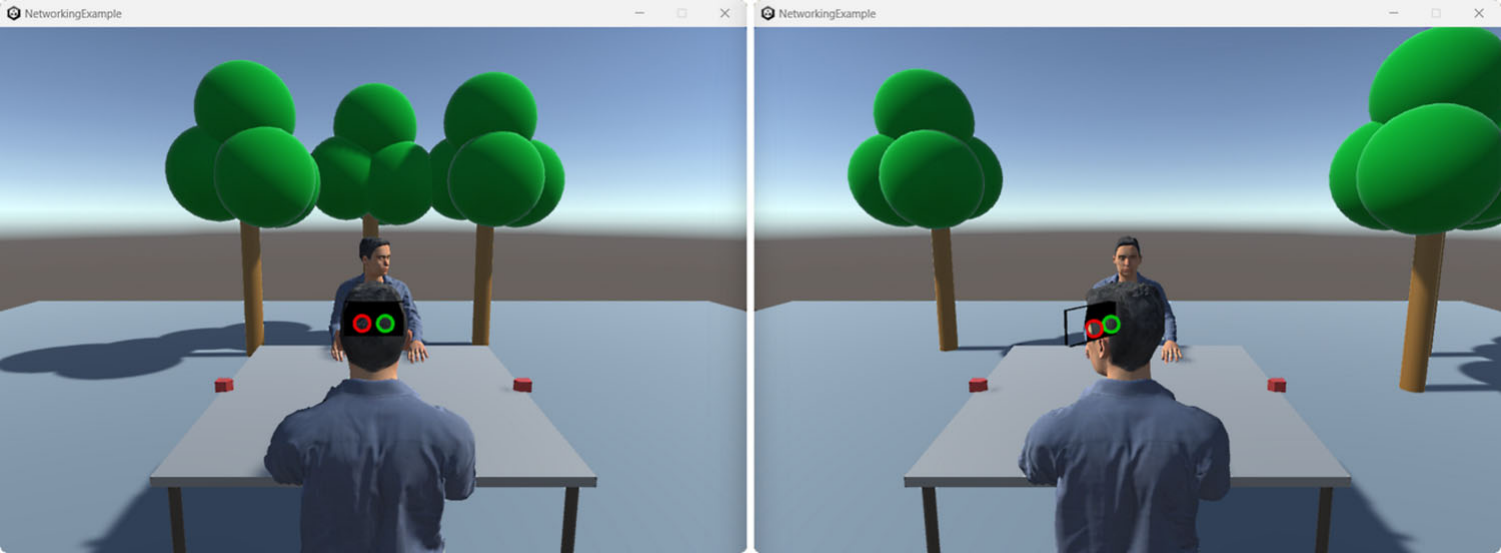
The same simulation is created and updated in two instances of the client software, each depicting the scene from the perspective of a character which is controlled via the mouse cursor (looking direction) and space bar (smiling). The example mimics a situation where the character is controlled to align with the known positions of a user’s eyes as in VR setups

Although the program currently acts as a host (with the server and client code enabled), the character sits in solitude as no other client has joined the table. To create a meeting of characters, several instances of the client software must connect to the server. With the server and one client running in the Unity Editor, another client can be run as a standalone program. To create a standalone client program, deactivate the server in the ‘Networking’ object before building the scene into a standalone program under *File* -$>{}$ *Build Settings* -$>{}$ *Build*. Again start the scene in the Unity Editor with the server and client enabled and run ‘NetworkingExample.exe’ in the newly created build folder. You will see two avatars in both windows representing, from their respective perspective, the self and the other avatar as shown in Fig. 4. Note how each user’s character visualizes eye positions as received from of a mock eye-tracking API to mimic working with VR tracking APIs. Now you can also see that pressing the space bar makes your character smile. Starting another instance of the built client program will result in three simulations each depicting three avatars. To use the environment entirely outside of the Unity Editor, you can either build a host version of the program where both the server and the clients are activated or build a version where only the server is activated, and all other objects in the scene may be deactivated. In total, there must be one server instance and there may be up to four client instances for the networked setup to work properly.

**Fig. 5:**
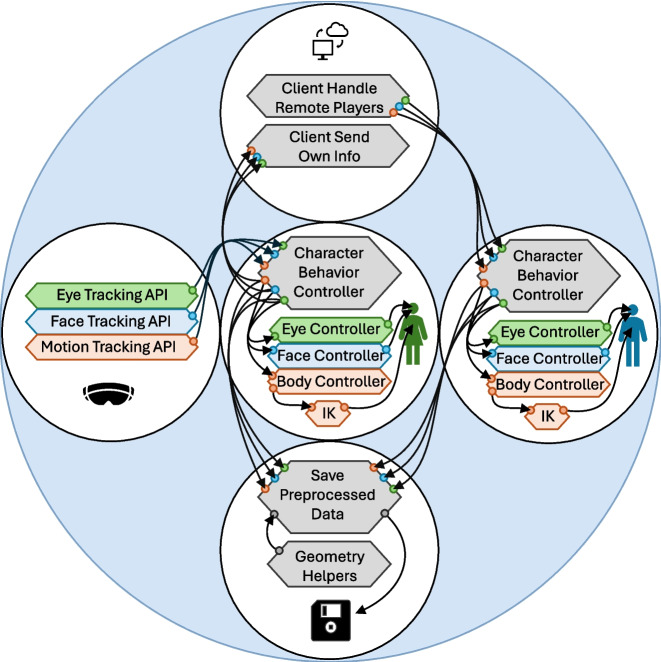
A closer look into the client software as it controls its own body illusion setup, sends its avatar’s state to the server and updates other users’ avatars along data received from the server. A center script $\textit{CharacterBehaviorController}$ is used on each character to allow for a flexible replacement of sensor APIs and character models and to organize data logging. IK: inverse kinematics, used to control a character’s posture based on positions and orientations of extremities.

Up to this point, the different instances of the client software are running on the same computer. This setup is sufficient to follow the software’s design but will not represent the typical use case of research with networked software. For a truly networked meeting, follow these steps: (1) ensure that at least two computers are connected to the same private local network, (2) decide which of the computers will run the server logic and obtain its local IP address (e.g., using cmd and typing ‘ipconfig’ on Windows), (3) copy the IP address to the field ‘Server IP’ in the *ClientStartNetwork* component on the Networking/Client object (replacing ‘localhost’), (4) save and again build programs representing the clients and either one server or host (5) copy the client program to the other computer(s) and (6) run the respective programs on each computer. It may additionally be required to allow the program’s networking communication through the firewall. As previously, each program window will now show the scene depicting the respective number of avatars from one’s own perspective but now running on different computers. If this setup is to be used for an extended period of time, consider assigning a static IP address to the server to prevent it from being assigned a new IP address during a research project.

### A detailed look at the information flow

Manipulating the software to construct one’s own experimental software will require more detailed knowledge of its organization. This tool follows general software guidelines but also tailors its code structure to the specific use case of experimental laboratory research (Evans, 2004; Fowler, 2018). As in other software, functionalities are implemented as modules which can be switched on and off without interfering with other parts of the code: In the control of avatars, sensor APIs can be seamlessly replaced; the body illusion setup can be run in isolation or with netcode enabled; logfile writers can be selected or removed based on specific research goals. By contrast, the code structure partly deviates from other software tools and code examples which are tailored towards the creation of entertainment software (e.g., https://github.com/oculus-samples/Unity-SharedSpaces) in its emphasis on more openly explicating the flow of information. Note that networked simulations often use modules which encapsulate data transfer between computers. For instance, developers can often simply tag objects as ‘networked’ for them to appear in the same position and orientation across computers, allowing to disregard underlying processes which implement the synchronization. While practical in several situations, such code encapsulation can interfere with researchers’ demands in controlling a diverse set of input data, implementing specific experimental variations and concisely integrating logfile writing functionalities.

Figure 5 more closely shows information flow in one instance of the client software in updating character states (i.e., after a connection to the server has been established and other users’ characters have been instantiated). Each hexagon represents a script (termed *class* in C#) in the example software. Arrows indicate that information is being transmitted between scripts, either when a script sends it to another script by calling a function on it or when a script inquires it from another script. A description on all scripts in the example software is given in the manual accompanying the software.

Each character – regardless of whether it represents one’s own avatar or another person’s avatar – is controlled by a center script named *CharacterBehaviorController* which holds all the information describing its state. It can similarly receive updates from VR sensors (transmitted through their respective APIs) when representing the user’s own avatar or from the script *ClientHandleRemotePlayers* (which receives updates from the server) when representing another user. Note that the example software contains mock versions of an EyeTracking API, FaceTracking API and BodyTracking API to mimic an implementation of networked software with VR hardware. By implementing standard data structures in the mock setup, transfer to a use with actual HMD APIs is typically straightforward. *CharacterBehaviorController* forwards character state data to the scripts *EyeController*, *FaceController* and *BodyController* which implement them on the character model, adapting them to a specific character models which may come with unique definitions for gaze directions, facial expressions or body postures. Since motion trackers in VR often do not capture the whole body but only the upper extremities (the head and hands), an additional inverse kinematics (IK) system (Aristidou & Lasenby, 2011) is typically implemented to control body postures. On the avatar representing the user, *CharacterBehaviorController* sends its state to the script *ClientSendOwnInfo* which sends it to the server. *CharacterBehaviorController* components of all active users send data to the logfile writers which store them directly or after additional preprocessing. When instantiating a new character as another user joins the network, *ClientHandleRemotePlayers* conveniently uses a pre-fabricated object definition where a character and required scripts are already assembled.

### Adapting the example software

Researchers creating their own social VR experimental software may leave the overall organization and a range of functionalities unaltered but will likely integrate new pieces of information which are exchanged among clients. The following shows how information is transmitted among clients by the example of exchanging character state data. Communication between instances of the client software follows the pattern of (1) a client sending data to the server, (2) the server relaying data to other clients (3) clients receiving data and updating their own simulation accordingly.

For every type of message which is to be exchanged, an empty form defining its contents (termed *broadcast* in Fish-Net networking) is defined. Here we create a broadcast for the character’s states and name it *currentPlayerState*. It holds the user’s ID in the network system, several positions (represented as Vector3) and orientations (represented as Quaternion) as well as a float value indicating the extent to which the character is smiling. The broadcast is stored in the script *MyBroadcasts* along with other broadcast definitions and is available to both the client and the server.
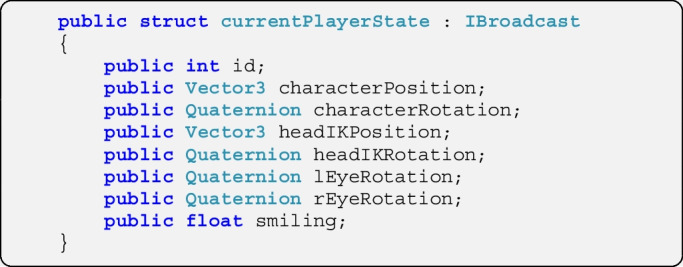


On the client in the script *ClientSendOwnInfo*, the pre-defined broadcast form is filled in every frame and is sent to the server. All relevant data are held by the own avatar’s *CharacterBehaviorController* script which is referenced here as *MyCharacterBehaviorController*. When sending data, developers can choose between using a ‘reliable’ and an ‘unreliable’ channel. Reliable messages are resent if the receiver does not confirm receiving the message (e.g., due to network packet loss). This technique is appropriate when sending messages which are sent rarely and/or trigger specific decisions such as the assignment to a specific seat on the table by the server. For data which are sent continuously such as characters’ states, unreliable messaging is typically more appropriate. Here, occasionally lost messages are not resent since a newer message likely reaches the receiver within a shorter period of time.
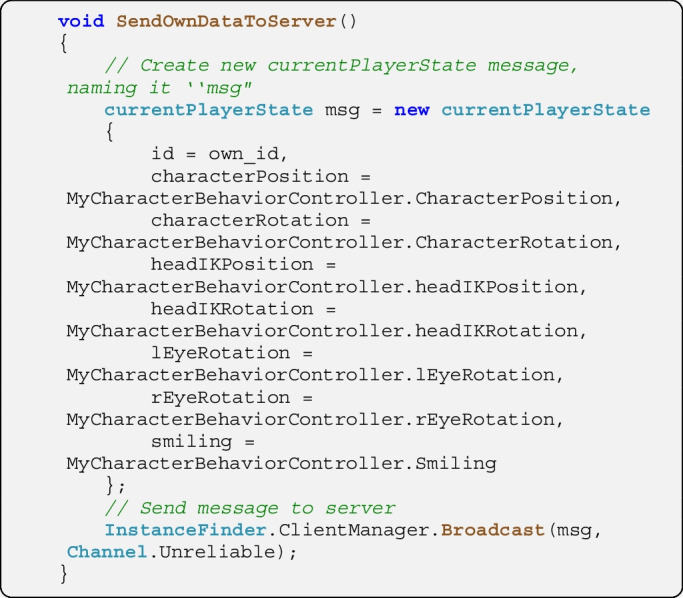


On the server in the script *ServerLogic*, the function *OnPlayerStateUpdate* is triggered when a message of the type *currentPlayerState* is received. The server script is also notified about the sender’s address (i.e., its *NetworkConnection*). Here the function merely relays the message to all other clients. The script could be extended to act upon the data in the message, e.g., to represent it for a spectator’s view of the simulation or to implement its own logfile writing.
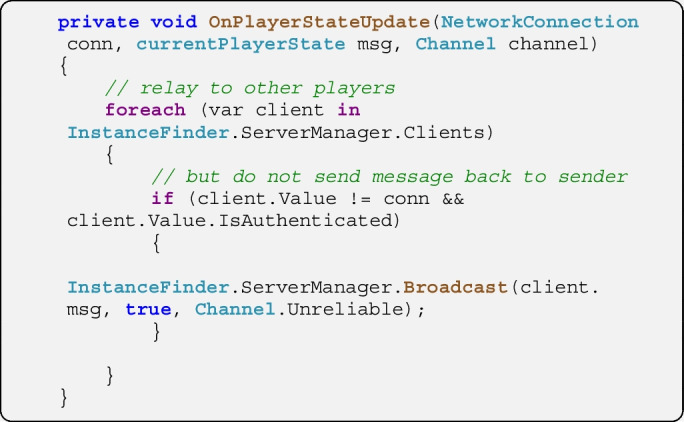


The relayed message is received on other clients. Here the function *OnPlayerStateUpdate* in the script *ClientHandleRemotePlayers* is triggered when a message of the type *currentPlayerState* is received. The script *ClientHandleRemotePlayers* keeps a list of characters representing other players (more specifically, their *CharacterBehaviorController* components) and fetches the avatar with the ID specified in the message. It forwards data to the respective *CharacterBehaviorController* which implements them.
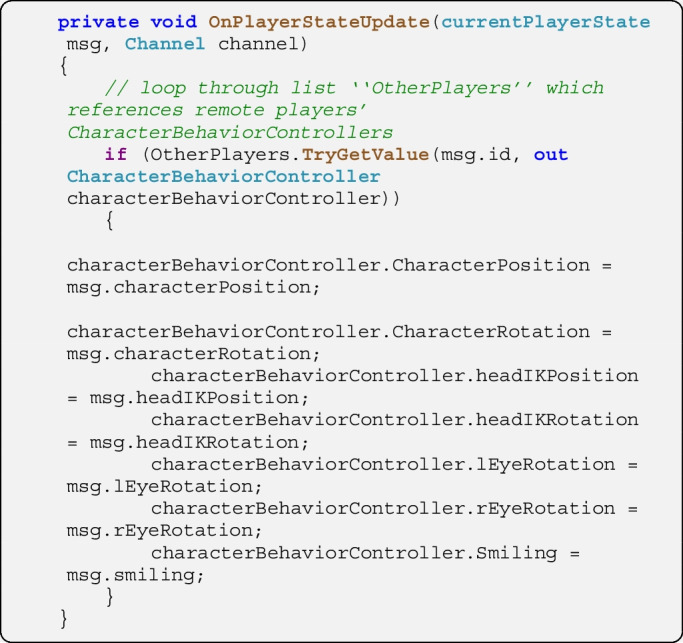


### Data collection and analysis

**Fig. 6:**
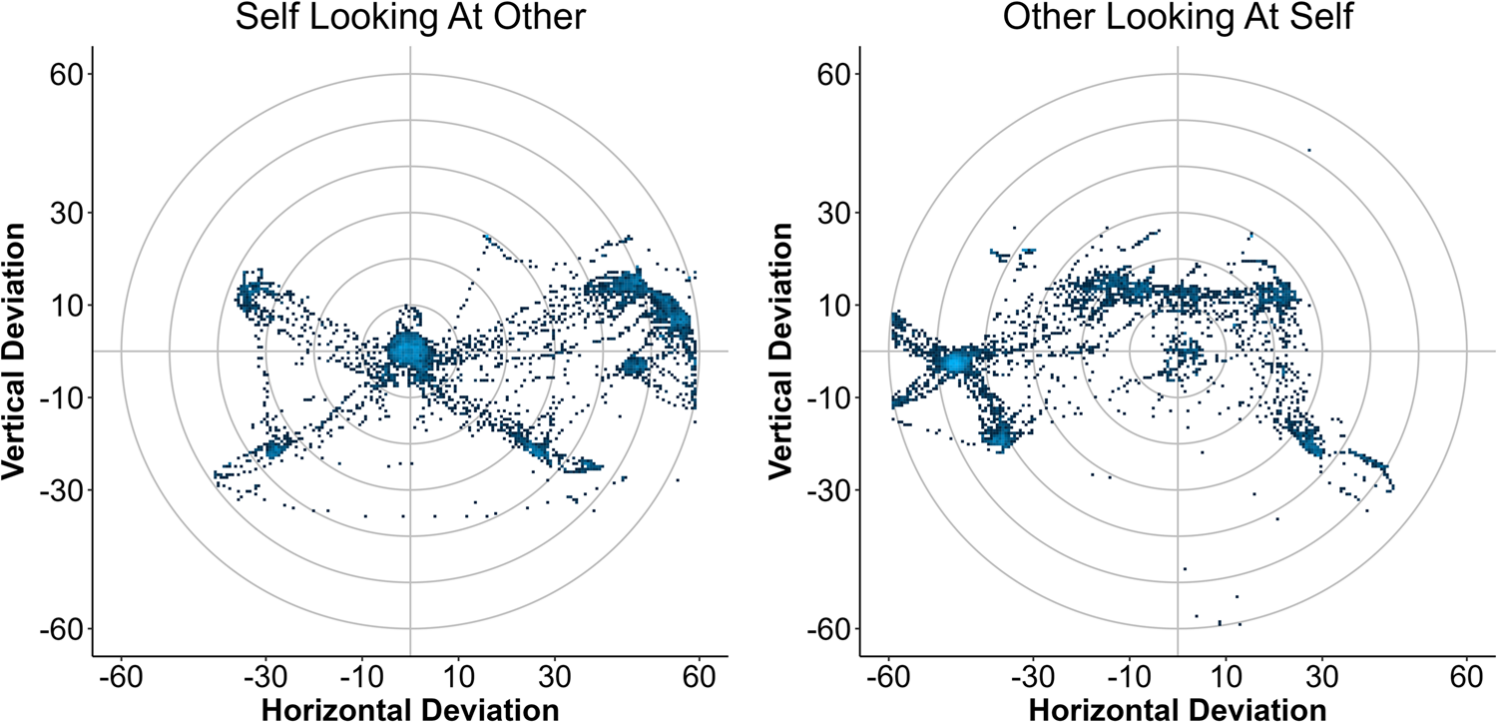
Each client computer stores data from its own as well as other users’ avatars in the same format. In addition to storing raw data, gaze data are stored after a transposition to represent deviations from pre-defined objects of interest in the horizontal and vertical direction. Here, data points in the center of the plot represent gaze towards the other avatar’s eyes

Data are written to disk in real-time on the client using logfile writer scripts. *SaveRawData* directly notes down data from all active *CharacterBehaviorController*, i.e., both for one’s own avatar and for all other characters. For analyses on gaze behavior, the data format stored by *SavePreprocessedData* may be more appropriate: Here, spatial gaze data are transposed using logic in *GeometryHelpers* to represent how each character gazes in the horizontal and vertical direction relative to another character’s eyes. Condensing data from 3D space onto 2D this way allows to continue data processing in parallel with monitor-based eye-tracking setups (e.g., implementing drift correction and region-of-interest analyses). Figure 6) shows gaze of the own avatar relative to a partner as well as gaze of the partner relative to the user’s own avatar. Similarly, as in left screen in Fig. 4, the own avatar mostly gazed towards the partner while the partner mostly gazed to its left. See the folder *ExampleData* for data and a corresponding R-script. Note how storing data on the client produces partly redundant files where the “self” in one file corresponds to the “other” in the other file, which allows assessing the agreement between simulations if needed.

### Integrate with virtual reality setup and extend

This software tool does not interact with VR hardware but closely mimics a social VR setup by implementing mock versions of motion-tracking, eye-tracking, and face-tracking APIs as commonly used with VR hardware. This design decision allows for the construction of a general software tool, which is independent from individual manufacturers’ APIs and furthermore helps to easily trace information flow while working on only a single computer. The tool is being used in our own laboratory when implementing and debugging novel features for social VR research. When integrating the tool with existing VR infrastructure, note that HMDs from different manufacturers may come with their own API but typically provide data in a similar form. For instance, eye gaze is typically represented as positions as well as looking directions of each eye in 3D space. Facial expressions are typically represented as a list of individual values decoding the activation of individual facial muscles or groups of muscles (e.g., raising the inner part of the left eyebrow, pulling the right lip corner etc.). Data available in different APIs can show close resemblance to or deviate more strongly from scientific categorizations of facial muscle activity such as the Facial Action Coding System (Canedo & Neves, 2019; Ekman & Rosenberg, 2005).

In order to transfer the software to a networked VR situation, the included mock APIs are replaced with the hardware’s actual APIs. The rest of the software can, in principle, remain unchanged. Depending on the employed hardware capabilities and research goals, researchers may wish to add an exchange of hand movements, finger movements and additional facial expressions along the routines shown here. Note that while the open-source inverse kinematics (IK) solution added in this software (*Fast IK*, https://github.com/ditzel/SimpleIK) implements a state-of-the art IK algorithm (Aristidou & Lasenby, 2011), other IK tools extend its functionality to allow for more natural and realistic body movements which are more directly tailored for VR use (e.g., *FinalIK*, http://root-motion.com/).

A common component of social VR setups which researchers may wish to implement is voice chat. While open-source tools for voice chat exist (e.g., https://webrtc.org/), a tool which can be easily connected with Fish-Net using a dedicated integration is Dissonance Voice Chat (https://placeholder-software.co.uk/, integration available at https://github.com/LambdaTheDev/DissonanceVoiceForFishNet). Voice chat tools add their own network layer on top of existing networks which, similar to other modular tools in networking, can make it difficult to follow the flow of information. This software therefore includes scripts in the folder *DissonanceTools* which more manually control voice chat to more flexibly design experimental software and harmonize the structure of data logging with the logging of behavior and gaze data. Voice chat can be complemented with real-time lip synchronization (lip-sync) which can be implemented using software that comes with a VR headset’s APIs (e.g., Oculus) or external software (e.g., https://github.com/hecomi/uLipSync).

The example software uses a character model created with the open-source tool *MakeHuman* (http://www.makehumancommunity.org/). While fully functional, other tools provide enhanced visual quality and more fine-grained control of facial expressions. In our own setup shown in Fig. 1, we use characters created using iClone Character Creator 4 (https://www.reallusion.com/character-creator/) with its *Headshot* plugin. When implementing personalized avatars, which more closely mimic each user’s physical appearance, note that character models must be distributed to all computers either by including them in a software build or by transmitting character data on the fly at the start of a social VR meeting (Rubo, 2024).

Replicating research from our laboratory (Son & Rubo, 2025) requires using the tool with higher-quality avatar models and to implement voice-chat along with corresponding logging of speaking behavior as described above. Such a setup is sufficient for detailed investigations into the properties of dyadic interaction behavior (e.g., speech–gaze interactions) or to assess attention biases such as eye gaze avoidance in socially anxious individuals (Caruana et al., 2021; Rubo & Munsch, 2024). Depending on the research design, additional events such as a display of instruction texts may be implemented along the procedure described in the Section“Adapting the example software". Other research projects may move beyond investigating conversations and include the assessment of behavior during collaborative problem-solving (CPS) tasks (Graesser et al., 2018; Sayadi et al., 2024). Analyses of speaking and gaze behavior are likewise central to understanding interaction behavior in CPS (Caruana et al., 2021; Nalepka et al., 2019; Schneider & Bryant, 2022; Schneider et al., 2018) and may be implemented in a similar fashion as in research on interaction behavior during conversations. By contrast, the display and logging of object manipulations across participants in a virtual scene again requires extending the software tool accordingly along above-mentioned procedure.

### Moving beyond the laboratory

The goal of this software is not to create a large-scale Metaverse application but specialized experimental software which deliver uncompromising social VR simulations in a controlled laboratory environment. Such controlled environments allow to tailor software to specific VR devices, reduce network latency to a minimum and administer additional laboratory measurements. Conducting research outside of laboratories comes with additional restrictions in hardware use and experimental control but may be the approach of choice in several research areas (Steed et al., 2023). Several steps are required to transition an application designed for internal laboratory use to an application which can be deployed in field research: (1) when network communication traverses the internet, the server must be reachable from outside of the local network. This can be realized by forwarding a specific port on the router to the server software which, in a laboratory environment, will often be handled by the IT administration team. If the server’s IP address should not be hard-coded into the client software to allow for changing IP addresses, a workaround is for the server software to write it to a static and protected file with a static address (see https://github.com/mariusrubo/Unity-StoreDataOnServer for examples on interacting with text files on the internet from within a Unity application). (2) While network communication inside of a protected local network may often not need additional security measures, communication over the internet may require authentication procedures and possibly encryption of behavioral and speech data. Fish-Net provides guidelines for the implementations of these techniques in its documentation. (3) While network communication in local networks typically features low network latency, internet communication may be associated with increased overall latency as well as latency spikes. To ensure visually smooth movements of characters, movements may be interpolated and extrapolated, which was not necessary in our own study (Son & Rubo, 2025) where computers communicated in a local network.

## Conclusion

Social Virtual Reality (VR) extends basic and applied psychological research in a range of fields and allows to develop novel and promising treatment applications. While several research questions can be addressed using commercially available social VR software, demands for flexible experimental control or secure data handling can call for the development of an in-house application. Here, I describe the architecture of such a setup along an example software for Unity where simulations on different computers are synchronized to create the impression of a shared situation with avatars sitting together on a table. The example software runs on an individual computer with no need for VR hardware but closely mimics a VR setup in its emulating of VR tracking APIs. The software features a single-user avatar control at its core – corresponding to a body illusion setup when combined with VR hardware – with networking as well as logfile writing functionalities added as modules. Avatars’ body movements are aligned to fit with a participant’s eye positions in order to allow for eye contact in the virtual environment. The structure of network communication emphasizes transparency over code brevity in order to implement a well-organized writing of data and to allow researchers to flexibly tailor scripts to their own demands. In logfile writing, gaze data is transposed to conform with 2D representations of gaze as in monitor-based eye-tracking research. The software focuses on aiding in creating social VR tools for the use in controlled research laboratory environments but can likewise serve as a starting point for tools that are intended to be used outside of a laboratory.

## Open practices statement

The software and shown example data are available at https://github.com/mariusrubo/SocialVRExample. The tool can be used freely for research purposes. No analyses were preregistered, as the tool is presented for demonstration purposes only.

## Data Availability

All data and materials are available at https://github.com/mariusrubo/SocialVRExample.

## Code Availability

Code is available at https://github.com/mariusrubo/SocialVRExample.
